# Case report: A novel case of COVID-19 triggered tumefactive demyelinating lesions in one multiple sclerosis patient

**DOI:** 10.3389/fnins.2023.1287480

**Published:** 2023-11-21

**Authors:** Jinghan Hu, Leiyun Huang, Zengyun Qiu, Yongzhen Liu, Kaiming Shen, Bin Tang, Jing Qian

**Affiliations:** ^1^Department of Neurology, People’s Hospital of Wenshan Prefecture, the Affiliated Hospital of Kunming University of Science and Technology, Wenshan, China; ^2^Medical College, Kunming University of Science and Technology, Kunming, China; ^3^Oftalmologia, People’s Hospital of Wenshan Prefecture, the Affiliated Hospital of Kunming University of Science and Technology, Wenshan, China; ^4^Department of Imaging, People’s Hospital of Wenshan Prefecture, the Affiliated Hospital of Kunming University of Science and Technology, Wenshan, China; ^5^Department of Respiratory Medicine, The First People’s Hospital of Yunnan Province, Kunming, China; ^6^The Affiliated Hospital of Kunming University of Science and Technology, Kunming, China

**Keywords:** multiple sclerosis, tumefactive demyelinating lesions, teriflunomide, SARS-CoV-2, COVID-19

## Abstract

The epidemic of COVID-19 is mainly manifested by respiratory symptoms caused by SARS-CoV-2 infection. Recently, reports of central nervous system diseases caused or aggravated by SARS-CoV-2 infection are also increasing. Thus, the COVID-19 pandemic poses an unprecedented challenge to the diagnosis and management of neurological disorders, especially to those diseases which have overlapping clinical and radiologic features with each other. In this study, a 31-year-old female patient had been diagnosed with relapsing–remitting multiple sclerosis (RRMS) initially and subsequently developed tumefactive demyelinating lesions (TDLs) following an infection with SARS-CoV-2. After immunotherapy (glucocorticoid pulses), a significant improvement was observed in her both clinical and radiological characteristics. The patient was started on disease-modifying therapy (DMT) with teriflunomide after cessation of oral glucocorticoids. Following two months of DMT treatment, the imaging follow-up revealed that the patient’s condition continued to deteriorate. This case was characterized by the transformation of a multiple sclerosis patient (MS) infected with SARS-CoV-2 into TDLs and the ineffectiveness of DMT treatment, which added complexity to its diagnosis and treatment. The case also gave us a hint that SARS-CoV-2 has a potential contributory role in inducing or exacerbating demyelinating diseases of the central nervous system that warrants further investigation.

## 1 Introduction

Multiple Sclerosis (MS) is a disease mediated by autoimmunity, which causes demyelination in the central nervous system. At present, the global median incidence of multiple sclerosis is 35.9 cases per 100,000 people (Walton et al., 2020). In China, the incidence rate is even lower, from 2016 to 2018, the incidence rate was 0.235 cases per 100,000 people per year (Tian et al., 2020). Therefore, it is considered a rare disease in China. MS can be classified into relapsing–remitting MS (RRMS), secondary progressive MS (SPMS), primary progressive MS (PPMS), or progressive-relapsing MS (PRMS) based on clinical phenotype and disease progression (Lublin et al., 2014). It is reported that patients with MS may develop tumefactive demyelinating lesions (TDLs), with an incidence rate of about (1 ~ 3)/1,000 in MS (Algahtani et al., 2017). TDLs, also known as tumefactive inflammatory demyelinating disorder (TID), are a rare form of central nervous system demyelination and are also related to autoimmunity (Altintas et al., 2012). Recently, some reports suggest that SARS-CoV-2 infection may trigger or exacerbate central nervous system demyelinating diseases such as MS (Fajgenbaum and June, 2020; Bellucci et al., 2021). However, no cases of MS transforming into TDLs have been reported following infection with the SARS-CoV-2 virus.

Here, we reported a novel case of a female patient with RRMS who developed TDLs after a SARS-CoV-2 infection. This case emphasizes the complexity and challenges encountered in diagnosing and managing tumefactive inflammatory demyelinating disorders in multiple sclerosis patients amidst a SARS-CoV-2 infection. Our case study also suggests that the SARS-Cov-2 virus may have contributed to demyelinating damages in the central nervous system (CNS).

## 2 Case description

**Figure 1:**
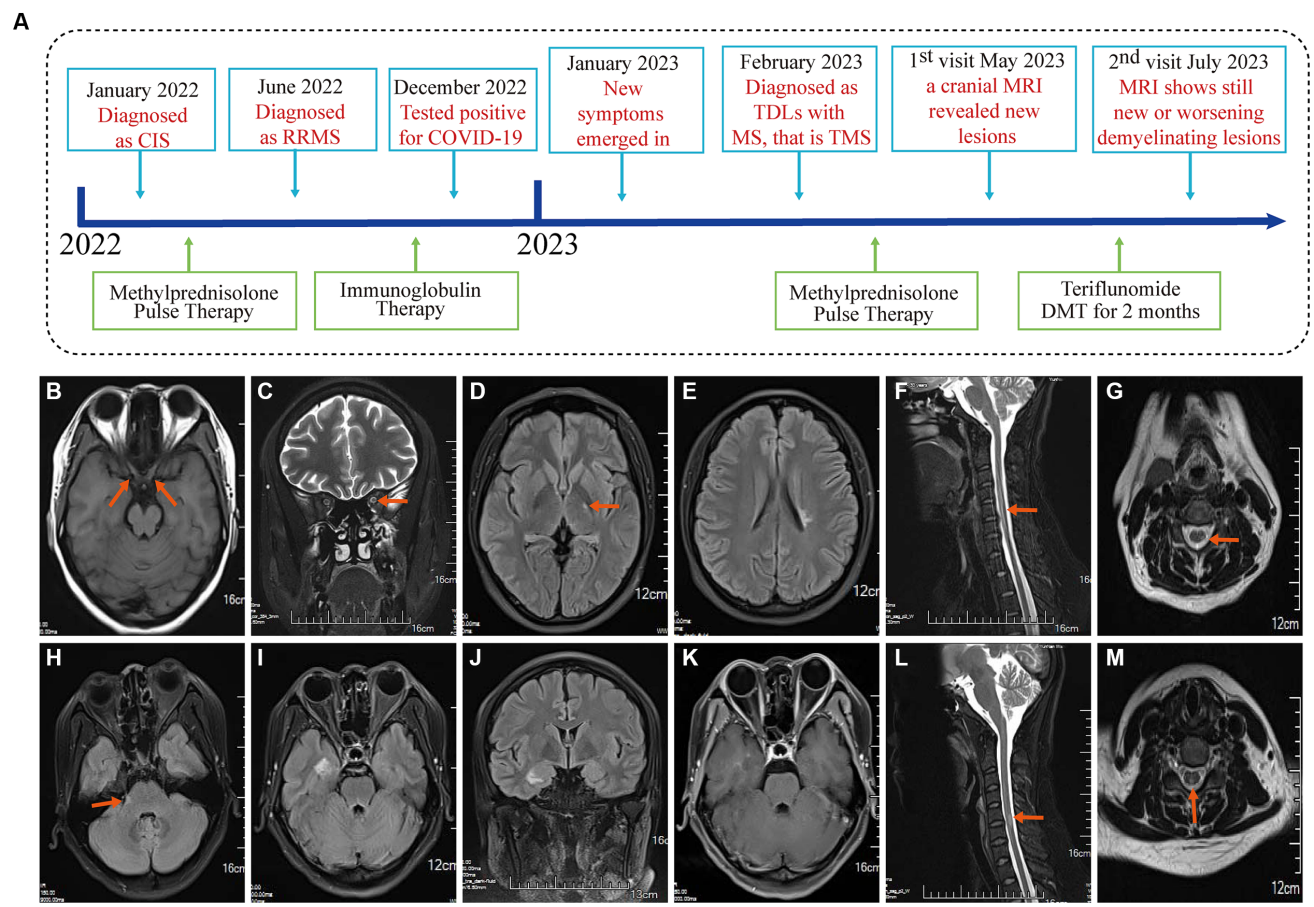
Event timeline for the case and MRI of the brain and spinal cord in January 2022 and June 2022. **(A)** The timeline showing the relevant events in the case. **(B,C)** T1WI and T2WI showed bilateral optic nerve enlargement and signal abnormalities. **(D,E)** The T2Flair displays small lesions located in the left posterior limb of the internal capsule and the cortical spinal tract area near the body of the left lateral ventricle. **(F,G)** T2WI revealed signal abnormalities in the cervical cord at the C3-6 vertebral plane. Diagnosed as a clinically isolated syndrome. **(H–J)** MRI of the brain and spinal cord in June 2022 with disease recurrence and T2Flair revealed new small lesions in the right cerebral peduncle, near the temporal horn of the right lateral ventricle, and in the hippocampal region. **(K)** T1WI-C shows right hippocampal lesion enhancement (active phase) in June 2022. **(L,M)** T2WI showed cervical spinal cord lesion shrinkage. Diagnosed with relapsing–remitting multiple sclerosis in June 2022.

The timeline (Figure 1A) shows the relevant events in this case from diagnosis as CIS to the follow-up.

### 2.1 Clinical features

On January 26, 2022, a 31-year-old female was admitted to the People’s Hospital of Wenshan Prefecture (Wenshan, Yunnan, China) due to blurred vision. Neurological physical examination (NPE): Vision is reduced in both eyes, with a significant decline in the left eye. Uncorrected visual acuity: VOD 0.08, VOS 0.06, Corrected visual acuity: VOD 4.8, VOS 4.8. There is a visual field defect in the central nasal side of the left eye, Abnormal pupillary reflex in the right eye, and present with a right-sided Marcus Gunn pupil sign. An optical coherence tomography (OCT) for both eyes, a visual evoked potential (VEP), and brainstem auditory evoked potential (BAEP) all returned normal results. MRI examination of the optic nerve, brain, and spinal cord was performed (Figures 1B–G). A cerebrospinal fluid (CSF) examination revealed positive oligoclonal bands, while serum oligoclonal bands, routine CSF tests, proteins, glucose, and chloride were all normal. There were no antibodies detected in either the cerebrospinal fluid or the serum for AQP4, MoG, GFAP, or MBP. The serum laboratory tests were unremarkable. It is suspected that the patient has clinically isolated syndrome (CIS). Then, she received pulse therapy of methylprednisolone sodium succinate 1 g/day, which was reduced by half every 3 days. Then, she was discharged with her vision significantly improved and clinical symptoms ameliorated. The steroid dosage was gradually reduced until it was discontinued.

On June 27, 2022, the patient was admitted to our hospital because of numbness in both lower limbs. Neurological physical examination (NPE): Decreased superficial sensation in both lower limbs, more pronounced in the right, with both lower limbs showing abnormal graphesthesia and position sense. The MRI of the brain and spinal cord revealed an increase in demyelinating lesions in the brain (Figures 1H–M). The results of the serum laboratory tests and the examination of the CSF were unremarkable. She has been diagnosed with RRMS and was treated with human immunoglobulin for 5 days, and her symptoms improved before discharge. She did not take any DMT-related drugs after discharge.

**Figure 2:**
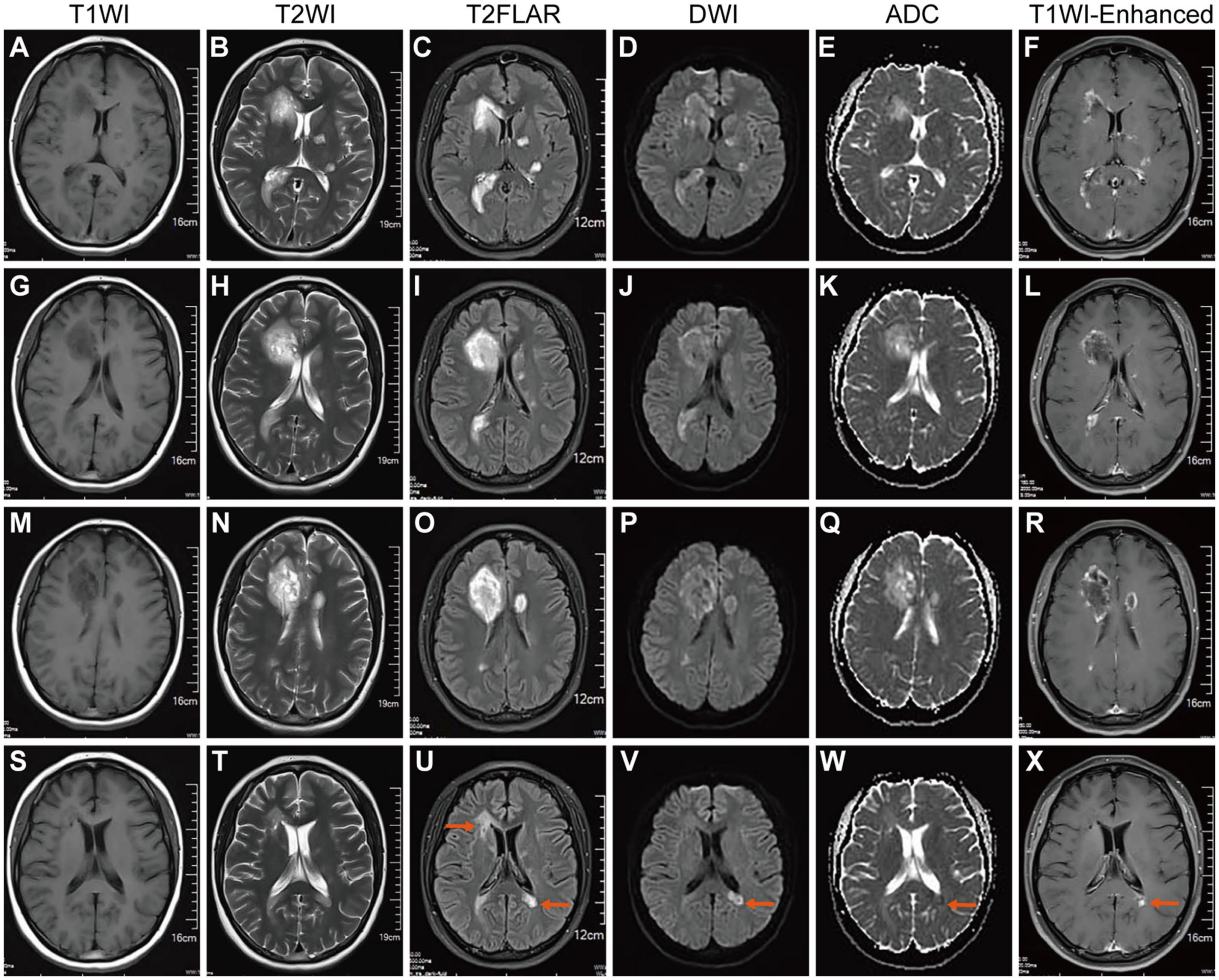
Brain MR on February 2023 after SARS-CoV-2 infection and in May 2023. **(A,G,M)** T1WI, **(B,H,N)** T2WI, and **(C,I,O)** T2 Flair showed multiple newly formed tumorous lesions around both ventricles and a small patchy lesion in the left basal ganglia region. **(D,J,P)** DWI showed high signal intensity from these lesions. **(E,K,Q)** ADC revealed a decreased signal at the lesion margin (with diffusion limitations). **(F,L,R)** T1WI-C revealed ring-shaped enhancement of tumorous lesions (active stage). Indicating intracranial TDLs. **(S–U)** After steroid pulse therapy, brain MR was rechecked on May 2023.T1WI, T2WI, and T2 Flair showed that intracranial tumorous lesions were smaller than before and another lesion appeared near the left lateral ventricle horn. **(V)** DWI revealed a markedly decreased signal around both ventricles and diffusion limitation was found near the left lateral ventricle horn. **(W)** ADC revealed a slightly decreased signal from the lesion near the left lateral ventricle horn. **(X)** T1WI-C revealed no enhancement, lesions around both ventricles liquefied, and the lesion near the left lateral ventricle horn enhanced (active stage).

On February 20, 2023, the patient was admitted to our hospital due to dizziness, and headache for one month. Neurological physical examination (NPE): Recent memory is reduced, with sequential forgetting. MMSE score (27 out of 30), and other neurological examinations show no abnormalities. The ratio of peripheral blood B cells to lymphocytes: is 31% (reference value: 5–18%). T cells in peripheral blood (CD3+, normal lymphocyte ratio). NK cells in peripheral blood (CD3-CD16+ or CD56+, normal lymphocyte ratio). Routine blood tests, erythrocyte sedimentation rate, liver and kidney function, cardiac enzymes, electrolytes, blood lipids, complement, serum immunoglobulins, thyroid function, tumor markers, ANA, ANCA, presurgical tests, coagulation function, BNP, PCT, and IL-6 were all normal. The pharyngeal test paper was negative for SARS-CoV-2 nucleic acid, and the serum SARS-CoV-2 antibody level was 102.63S/CO (Positive≥1S/CO), and IgM(−). Electrocardiograms and electroencephalograms were normal. Chest, abdominal, and abdominal CTs were all normal. An enhanced MRI of the brain and spinal cord (Figures 2A–R) revealed multiple speckled, mass-like nodular shadows in the right cerebellar hemisphere, left temporal lobe, left basal ganglia, periventricular white matter, right frontal lobe, and right portion of the genus and splenium of the corpus callosum. Upon enhancement, these showed ring-like, nodular, and peripheral striated flocculent-open ring-like enhancements. Additionally, there were patchy shadows within the spinal cord at the levels of C3-4 and C5-6 intervertebral spaces. Visual fields and ocular coherence tomography (OCT) were normal in both eyes. The analyses of CSF revealed damage to the blood–brain barrier (QAlb CSF/Serum 6.26 × 10^3^) and the presence of CSF-restricted IgG oligoclonal bands (Pattern II). There were no demyelinating antibodies in CSF or serum (AQP4, MOG, GFAP, and MBP) in the central nervous system (CNS). CSF SARS-CoV-2 antibody (magnetic particle chemiluminescence method) test showed IgG(+) and IgM(−). However, CSF next-generation sequencing (NGS, DNA + RNA) was negative, which did not rule out the possibility of recent intracranial SARS-CoV-2 infection. A diagnosis of TDLs with multiple sclerosis that is tumefactive multiple sclerosis (TMS) was ultimately diagnosed in this case. The patient refused a brain biopsy recommended to distinguish from brain tumors.

### 2.2 Diagnosis and differential diagnosis

Based on the 2017 McDonald criteria for diagnosing multiple sclerosis: The patient experienced more than 2 clinical attacks and had evidence of more than 2 lesions, thus being diagnosed with MS. Imaging evaluations indicated that the patient developed TDLs lesions.

#### Differential diagnosis

(1) Virus test: Three times of serum HIV test were negative. CSF toxoplasma antibodies, herpes simplex virus (II) antibodies,rubella virus antibodies, cytomegalovirus antibodies and EB virus antibodies(−). CSF pathogen mNGS sequencing was negative, which excludes other viruses that can induce acute episodes of CNS demyelination, such as ZikV and ChikV (Alves-Leon et al., 2018; Rueda-Lopes et al., 2021).
(2) Tests for AQP4, MOG, GFAP, and MBP in both CSF and serum were negative, ruling out NMOSD-related diseases. Multiple sclerosis is characterized by type II blood–brain barrier disruption, as evidenced by oligoclonal bands in the CSF.
(3) Autoimmune antinuclear antibody and erythrocyte sedimentation rate were both normal, excluding autoimmune encephalitis.
(4) Serum tumor markers and thoracoabdominal CT excluded paraneoplastic syndrome.
(5) The patient refused a brain biopsy recommended to rule out brain tumors. However, based on the significant improvement in imaging of the primary tumor-like lesions after two months of steroid treatment during the acute phase and the magnetic resonance spectroscopy examination, we temporarily excluded the possibility of a brain tumor.

The patient’s laboratory test results are shown in Table 1.

**Table 1:** The Laboratory tests of patient.

Times	Project title	Inspection content (Reference range)	Results
January 26, 2022	CSF	pressure(mmH2O) protein(0.15–0.45 g/L) glucose(2.5–4.5 mmol/L) chlorine(120–132 mmol/L) white blood cell(0–10 × 10^6^/L) Immunoglobulin G(10–30 mg/L) Coloration	170 0.251 2.79 126.9 10 32.1 clear and bright
	Blood biochemical	ANA、ANCA、ESCR、Thyroid function、white blood cells、CRP、PCT、IL-6、HIV、HCV、HBV、TP-Ab	normal
	Central nervous system demyelinating antibody	AQP4, MOG, GFAP, and MBP (CSF + Serum)	(−)
	Oligoclonal band CSF-restricted IgG ligoclonal bands	CSF、Serum (Pattern II)	(+) (−)
June 27, 2022	Blood biochemical	ANA、ANCA、ESCR、thyroid function、white blood cells、CRP、PCT、IL-6、HIV、HCV、HBV、TP-Ab	normal
	Tumor markers	Ca125、Ca153、Ca199、Ca724、Ca211、CEA、AFP、NSE	(−)
February 20, 2023	Peripheral blood lymphocyte subsets	Peripheral blood T-lymphocyte subsets (percentage)	normal
		Peripheral Blood NK Cells (CD3-CD16+ or CD56+)NK cells/lymphocytes (adult||7–40%)	9%
		Peripheral blood B-lymphocytes (CD3-CD19+), B-cells/lymphocytes (adult||5–18%)	31% ↑
	CSF	pressure(mmH2O) protein(0.15–0.45 g/L) glucose(2.5–4.5 mmol/L) chlorine(120-132 mmol/L) white blood cell(0–10 × 10^6^/L) Immunoglobulin G(10-30 mg/L) Coloration	175 0.332 3.19 127.8 2 31.8 clear and bright
	CSF mNGS	Pathogen(DNA + RNA)	(−)
	Oligoclonal band CSF-restricted IgG ligoclonal bands	CSF、Serum (Pattern II)	(+) (−)
	Central nervous system demyelinating antibody	AQP4, MOG, GFAP, and MBP (CSF + Serum)	(−)
	Serum&CSF SARS-CoV-2 antibody test	Serum:IgG CSF:IgG Serum:IgM CSF:IgM	(+) (−)
	Blood biochemical	ANA、ANCA、ESCR、thyroid function、white blood cells、CRP、PCT、IL-6、HIV、HCV、HBV、TP-Ab	normal

## 3 Patient’s history of COVID-19 vaccination and the evidence of SARS-CoV-2 infection influenced CNS

She received two doses of the inactivated novel Coronavirus vaccine (Vero cell) on June 3 and June 26, 2021, with no adverse reactions during the vaccination period and no particular medical history. On December 21, 2022, the patient experienced headaches, fever, generalized fatigue, and myalgia. On December 24, a throat swab test revealed a positive result for the nucleic acid test for SARS-CoV-2. During the acute phase of SARS-COV-2 infection, the patient highest body temperature was 38.7°C, and the SPO2 was 96%. Her symptoms improved after ibuprofen treatment. On January 10, 2023, the patient developed blurred vision, dizziness, and headaches, with the headaches manifesting as moderate to severe paroxysmal throbbing in bilateral temporal regions.

Following SARS-COV-2 infection and on the third times hospital admission, CSF and serum SARS-CoV-2 antibody (magnetic particle chemiluminescence method) test showed positive for IgG and negative for IgM.

## 4 Therapeutic interventions, follow-up and outcomes

### 4.1 Therapeutic interventions

A diagnosis of TDLs with multiple sclerosis was ultimately diagnosed in this case. As part of the treatment plan, the patient received pulse therapy with methylprednisolone sodium succinate 1 g/day, which was halved every 3 days and then tapered to 60 mg prednisone acetate orally/day, with a 5-mg reduction each week. After treatment, the patient’s vision, dizziness, and headaches significantly improved, and his recent memory gradually improved.

An enhanced MRI of the head on May 2, 2023 (Figures 2S–X), showed that the multiple TDLs had become smaller than before, some lesions appeared to soften, and new lesions were found under the left lateral ventricle triangle area, with limited diffusion and enhancement. It was discovered that she had new active lesions associated with MS. It was recommended that the patient begin taking teriflunomide again to initiate DMT modification therapy to reduce relapses.

### 4.2 Follow-up and outcome

**Figure 3:**
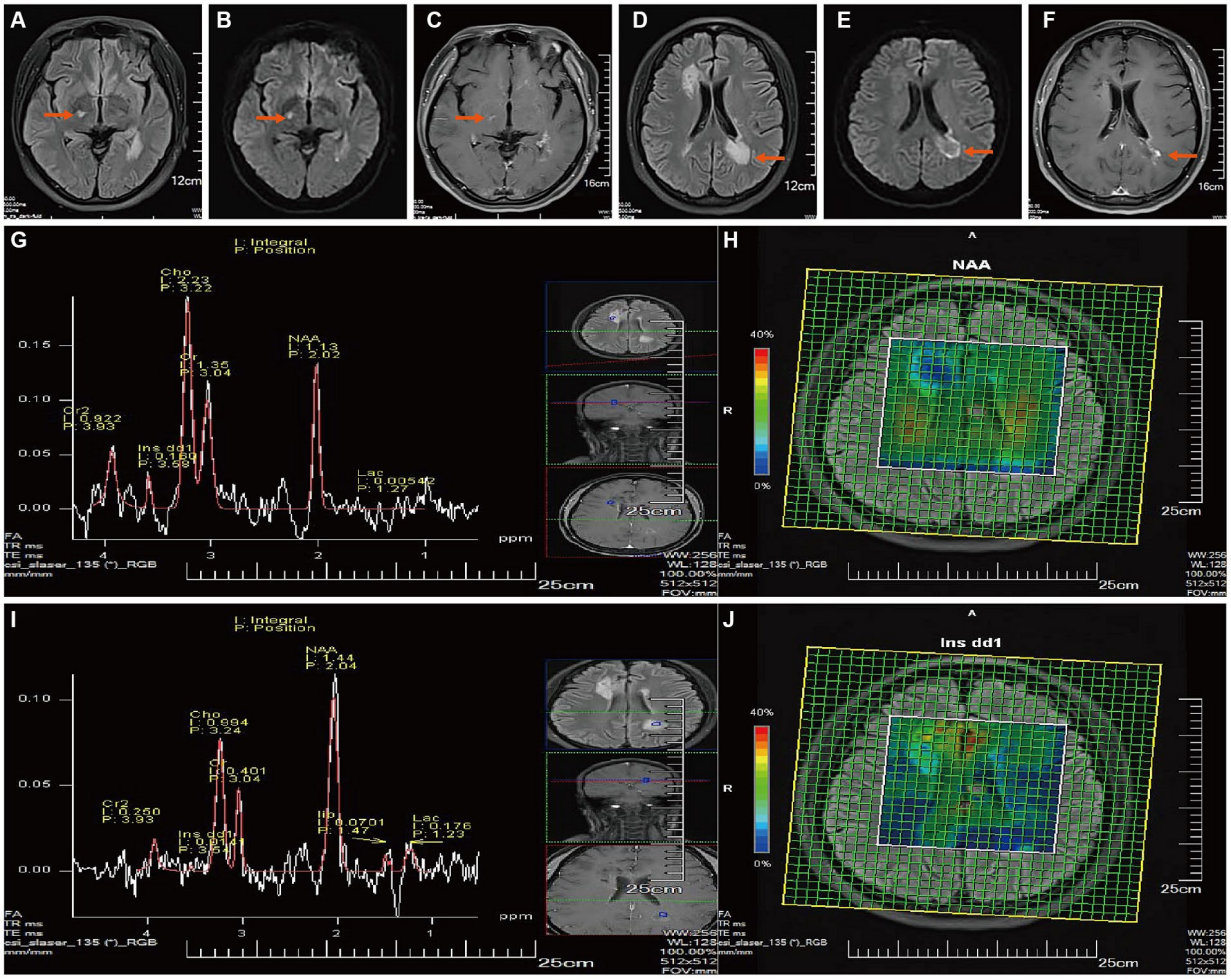
Follow-up Brain MR on July 2023. **(A,D)** After two months of treatment with Teriflunomide DMT: T2Flair revealed a new right thalamic lesion, the lesion near the apex of the left lateral ventricle had enlarged compared to the previous image, and the remaining intracranial lesions had shrunk and softened compared to before. **(B,E)** DWI showed high signals. **(C,F)** T1WI-C revealed spotty enhancement in the right thalamus and open-ring enhancement near the left lateral ventricle (active phase). [**(G, I)**, TE = 135 ms] MRS showed that the demyelinating lesion near the right anterior horn of the lateral ventricle is in the non-active phase **(G)**, Cho peak is elevated, and the NAA peak is slightly reduced, Cho/NAA ratio: 1.97; he demyelinating lesion near the left apex of the lateral ventricle is in the active phase **(I)**, the Cho peak is mildly elevated, and the NAA peak is mildly reduced, Lip and Lac peaks can be observed, Cho/NAA ratio: 0.69.Metabolite imaging CSI **(H,J)** shows that most of the lesion near the right anterior horn of the lateral ventricle, and a small part of the lesion’s center near the left posterior horn of the lateral ventricle are in the non-active phase (decreased NAA, increased Ins).

After two months of DMT medication, the enhanced MRI + MRS of the head on July 9, 2023 (Figures 3A–J) revealed that although the tumefactive lesion in the right frontal lobe of the patient’s brain had diminished and partially softened, a new lesion was found in the right thalamus and the lesion next to the triangle-vertex-posterior angle of the left lateral ventricle was larger than the previous film, and the enhanced group-like open-loop enhancement next to the left lateral ventricle was in the active phase. The MRS showed demyelinating lesions in the right frontal lobe and adjacent to the left lateral ventricle. The peripheral area next to the left lateral ventricle was an active lesion.

To sum up, there was no significant improvement in the MS lesions after the patient received modified treatment with teriflunomide and new lesions that transition into TDLs were identified. Unfortunately, the patient in this study refused further treatment and readmission.

## 5 Discussion and conclusion

This novel clinical case describes a patient with RRMS who developed TDLs after a SARS-CoV-2 infection and failed to suppress disease progression after 2 months of treatment with teriflunomide DMT. According to the patient’s medical history and treatment response, the conversion of MS to TDLs may be related to the following factors:

Firstly, the patient had a pre-existing MS condition before being infected with SARS-CoV-2, with a high immune sensitivity and susceptibility, and SARS-CoV-2 infection triggered a relapse or transition of MS (Bellucci et al., 2021; Macdougall et al., 2022). This suggests that SARS-CoV-2 infection may act as an environmental trigger for MS exacerbation or conversion. The mechanism may be related to SARS-Cov-2’s ability to activate the body’s immune response, including both the innate and adaptive responses (Li et al., 2020; Macdougall et al., 2022). On the one hand, by inducing an immune response against SARS-Cov-2, a large number of cytokines and chemokines (such as IL-6, IL-17, and IFN-γ) can spread throughout the body, causing a cytokine storm that can damage or demyelinate central nervous tissue (Fajgenbaum and June, 2020). The Cytokine storms and peripheral hyperinflammatory states may indirectly lead to impaired permeability of the blood–brain barrier (Huang et al., 2020). For example, mice infected with influenza A showed significant increases in LI-6, IL-1β, and TNF-α six days after vaccination, and increased vascular permeability was seen in the lungs and brain. (Wang et al., 2010). Therefore, the virus does not invade the brain directly, but may indirectly disrupt the blood–brain barrier, promoting the entry of inflammatory cells and molecules into the brain. On the other hand, the Cytokine storm caused by coronavirus infection may directly induce nerve damage. As is reported, SARS-CoV infection was found to trigger IL-6 expression in neurons and astrocytes in hACE2 mice model (Netland et al., 2008). In the study of neuroinflammation induced by SARS-CoV-2 spike glycoprotein S1 in BV-2 microglia, SARS-CoV-2 can trigger IL-6 expression in microglia (Olajide et al., 2022). Recent studies have shown that in the context of MS, microglia with pro-inflammatory effects can worsen and demyelinate MS by producing a variety of inflammatory cytokines (Colonna and Butovsky, 2017; Radandish et al., 2021). In addition, B cells may be involved in the cytokine storm caused by SARS-CoV-2 infection (Fajgenbaum and June, 2020), and may be involved in the production of autoantibodies against neural antigens and may enhance MS’s autoimmune response. The patient in this case had a significantly increased B/T lymphocyte ratio in peripheral blood (31%), indicating active B cell proliferation., and the increase of B cells detected in peripheral blood, in this case, may be related to this factor.

As a result of the cytokine storm that is caused by the SARS-Cov-2 infection, it may directly or indirectly worsen multiple sclerosis symptoms, especially demyelination. In the future, clinical treatment and research of demyelinating diseases, it is also necessary to further strengthen the understanding of the cytokine storm produced after SARS-CoV-2 infection, to facilitate personalized patient treatment.

Secondly, SARS-CoV-2 can directly enter the brain through the olfactory pathway. According to a previous study, the HCov-OC43 Coronavirus can be transported to the olfactory bulb via retrograde axons and spread to the hippocampus and other brain structures (Dube et al., 2018). K18-hACE2 mice infected with SARS-CoV-2 demonstrated similar neuronal transmission, with infectious viruses identified at 6 dpi in several brain regions, including the olfactory bulb, cerebral cortex, caudate/putamen, thalamus, hypothalamus, and ventral striatum (Zheng et al., 2021). These evidence suggests that the coronavirus can reach the intracranial through retrograde infection. When SARS-CoV-2 enters the brain, it can infect neurons and glial cells such as astrocytes and oligodendrocytes, leading to demyelination. This may cause direct viral damage to the myelin sheath and axons, as well as induce neuroinflammation and neurodegeneration. Viral infection may also activate T cells and autoantibodies against neural tissue by molecular mimicry, aggravating MS symptoms (Macdougall et al., 2022).

In summary, based on the patient’s medical history progression, we believe manifestations in the patient’s central nervous system are due to post-infection inflammatory reactions caused by SARS-CoV-2 and the long COVID syndrome. After the remission period of a MS flare-up, patient’s daily life was completely unaffected. During this time, she did not take any DMT medications, so there was no possibility of immune interference inducing a transition to TDLs. Shortly after infection with SARS-CoV-2, she quickly developed CNS symptoms, and imaging also confirmed the exacerbation of MS. Because the patient did not take specific medications to inhibit viral transcription (such as Pxlovid/Alzulfidine), viruses could enter the brain through the blood–brain barrier, and infection would activate microglia and astrocytes, releasing inflammatory mediators into the brain. The production of viruses in the body may change the microenvironment of CNS. This may aggravate demyelination, increase MS activity or progression, and cause new or worsening demyelinating lesions (Li et al., 2020; Macdougall et al., 2022). Through literature review, we found that many patients with central system diseases experienced worsening and progression of their conditions after contracting SARS-CoV-2 (Bellucci et al., 2021; Macdougall et al., 2022). This supported that under the influence of the virus or viral proteins, the human immune system can produce abnormal responses, leading to attacks on CNS. The Inflammatory response and inflammatory factors also enhance the demyelination response of CNS. Therefore, we propose that the case reported in this paper may be the result of a complex interaction between SARS-CoV-2 infection and MS. This provides clinical evidence that can be used in future basic research.

Currently, no specific treatment experience is available for patients with MS who develop TDLs as a result of contracting SARS-CoV-2. Clinical symptoms of the patient were largely alleviated after immunotherapy (glucocorticoid pulses) was administered. The patient was treated with teriflunomide administered as DMT. Teriflunomide was selected for the following reasons: As Teriflunomide was the first DMT drug approved in China in 2018, it is relatively easy for patients and diagnosis and treatment institutions in remote areas of western China to obtain Teriflunomide due to geographical and medical resource constraints. Further, Teriflunomide has been added to the list of special disease medical insurances in China. This will reimburse most of the costs and reduce the financial burden on patients. Finally, Capone et al. demonstrated, through a literature review, that teriflunomide should not be discontinued in MS patients infected with SARS-CoV-2. The treatment may even be safe in patients with severe complications who develop SARS-CoV-2-associated pneumonia and require hospitalization (Capone et al., 2021). It was, however, discovered two months after receiving DMT that the patient had continued to experience deterioration of demyelinating lesions and that even new lesions were developing. Thus, teriflunomide treatment did not control the transformation of our patient’s TDLs, suggesting that teriflunomide might be ineffective as a disease-modifying treatment for tumefactive multiple sclerosis (TMS) patients.

If TDLs appear in MS patients infected with the Coronavirus, we believe that it is critical to closely monitor and evaluate the patient’s response to DMT and, if necessary, switch to a more targeted medication as soon as possible. For instance, when MS patients have TDLs with spatial or temporal dissemination characteristics, seniltimod, oftulizumab, natalizumab, or alenzumab are recommended, which are highly effective in reducing recurrences. It has been demonstrated that monoclonal antibodies may be more effective than immunosuppressive drugs at removing B and T lymphocytes causing neuroinflammation, without adversely affecting normal cells (Yednock et al., 1992; Hauser et al., 2008).

This case report still has some limitations. As we reported on a rare case, with a small sample size, which cannot comprehensively reflect MS patients transitioning to TDLs due to SARS-CoV-2 infection or exacerbation. Moreover, the patient’s drug selection was limited by objective factors. The follow-up period in this case was relatively short, making it impossible to evaluate the long-term course. In the future, more cases and mechanistic studies will need to be conducted. However, to some extent, this report provides clinical reference points and offers suggestions for future research.

In summary, this case is the first report of a rare case of extensive TDLs in an MS patient after SARS-CoV-2 infection.

## Data availability statement

The original contributions presented in the study are included in the article/supplementary material, further inquiries can be directed to the corresponding author.

## Ethics statement

The studies involving humans were approved by The Ethics Committee of People’s Hospital of Wenshan Prefecture. The studies were conducted in accordance with the local legislation and institutional requirements. The participants provided their written informed consent to participate in this study. Written informed consent was obtained from the individual (s) for the publication of any potentially identifiable images or data included in this article.

## Author contributions

JH: Formal analysis, Writing – original draft. LH: Formal analysis, Writing – original draft. ZQ: Data curation, Investigation, Writing – review & editing. YL: Data curation, Writing – review & editing. KS: Data curation, Writing – review & editing. BT: Data curation, Writing – review & editing. JQ: Writing – review & editing, Supervision.

## References

[ref1] Algahtani H. Shirah B. Alassiri A. (2017). Tumefactive demyelinating lesions: a comprehensive review. Mult. Scler. Relat. Disord. 14, 72–79. doi: 10.1016/j.msard.2017.04.003, PMID: 28619436

[ref2] Altintas A. Petek B. Isik N. Terzi M. Bolukbasi F. Tavsanli M.. (2012). Clinical and radiological characteristics of tumefactive demyelinating lesions: follow-up study. Mult. Scler. 18, 1448–1453. doi: 10.1177/1352458512438237, PMID: 22419670

[ref3] Alves-Leon S. V. Lima M. D. R. Nunes P. C. G. Chimelli L. M. C. Rabelo K. Nogueira R. M. R.. (2018). Zika virus found in brain tissue of a multiple sclerosis patient undergoing an acute disseminated encephalomyelitis-like episode. Mult. Scler. J. 25, 427–430. doi: 10.1177/1352458518781992 30226115

[ref4] Bellucci G. Rinaldi V. Buscarinu M. C. Renie R. Bigi R. Pellicciari G.. (2021). Multiple sclerosis and Sars-CoV-2: has the interplay started? Front. Immunol. 12:755333. doi: 10.3389/fimmu.2021.755333, PMID: 34646278 PMC8503550

[ref5] Capone F. Motolese F. Luce T. Rossi M. Magliozzi A. Di Lazzaro V. (2021). Covid-19 in teriflunomide-treated patients with multiple sclerosis: a case report and literature review. Mult. Scler. Relat. Disord. 48:102734. doi: 10.1016/j.msard.2020.102734, PMID: 33429305 PMC7836732

[ref6] Colonna M. Butovsky O. (2017). Microglia function in the central nervous system during health and neurodegeneration. Annu. Rev. Immunol. 35, 441–468. doi: 10.1146/annurev-immunol-051116-052358, PMID: 28226226 PMC8167938

[ref7] Dube M. Le Coupanec A. Wong A. H. M. Rini J. M. Desforges M. Talbot P. J. (2018). Axonal transport enables neuron-to-neuron propagation of human coronavirus Oc43. J. Virol. 92, 8–9. doi: 10.1128/JVI.00404-18, PMID: 29925652 PMC6096804

[ref8] Fajgenbaum D. C. June C. H. (2020). Cytokine Storm. N. Engl. J. Med. 383, 2255–2273. doi: 10.1056/NEJMra2026131, PMID: 33264547 PMC7727315

[ref9001] Hauser S. L. Waubant E. Arnold D. L. Vollmer T. Antel J. Fox R. J.. (2008). B-cell depletion with rituximab in relapsing-remitting multiple sclerosis. N. Engl. J. Med. 358, 676–688. doi: 10.1056/NEJMoa0706383 18272891

[ref9] Huang X. Hussain B. Chang J. (2020). Peripheral inflammation and blood–brain barrier disruption: effects and mechanisms. CNS Neurosci. Ther. 27, 36–47. doi: 10.1111/cns.13569 33381913 PMC7804893

[ref10] Li X. Geng M. Peng Y. Meng L. Lu S. (2020). Molecular immune pathogenesis and diagnosis of Covid-19. J Pharm Anal 10, 102–108. doi: 10.1016/j.jpha.2020.03.001, PMID: 32282863 PMC7104082

[ref11] Lublin F. D. Reingold S. C. Cohen J. A. Cutter G. R. Sorensen P. S. Thompson A. J.. (2014). Defining the clinical course of multiple sclerosis: the 2013 revisions. Neurology 83, 278–286. doi: 10.1212/WNL.0000000000000560, PMID: 24871874 PMC4117366

[ref12] Macdougall M. El-Hajj Sleiman J. Beauchemin P. Rangachari M. (2022). Sars-CoV-2 and multiple sclerosis: potential for disease exacerbation. Front. Immunol. 13:871276. doi: 10.3389/fimmu.2022.871276, PMID: 35572514 PMC9102605

[ref13] Netland J. Meyerholz D. K. Moore S. Cassell M. Perlman S. (2008). Severe acute respiratory syndrome coronavirus infection causes neuronal death in the absence of encephalitis in mice transgenic for human Ace2. J. Virol. 82, 7264–7275. doi: 10.1128/JVI.00737-08, PMID: 18495771 PMC2493326

[ref14] Olajide O. A. Iwuanyanwu V. U. Adegbola O. D. Al-Hindawi A. A. (2022). Sars-CoV-2 spike glycoprotein S1 induces Neuroinflammation in Bv-2 microglia. Mol. Neurobiol. 59, 445–458. doi: 10.1007/s12035-021-02593-6, PMID: 34709564 PMC8551352

[ref15] Radandish M. Khalilian P. Esmaeil N. (2021). The role of distinct subsets of macrophages in the pathogenesis of Ms and the impact of different therapeutic agents on these populations. Front. Immunol. 12:667705. doi: 10.3389/fimmu.2021.667705, PMID: 34489926 PMC8417824

[ref17] Rueda-Lopes F. C. da Cruz L. C. H. Fontes F. L. Herlinger A. L. da Costa Ferreira Junior O. de Aguiar R. S.. (2021). Clinical and magnetic resonance imaging patterns of extensive chikungunya virus–associated myelitis. Journal of Neuro Virology 27, 616–625. doi: 10.1007/s13365-021-00962-4, PMID: 34227044

[ref18] Tian D. C. Zhang C. Yuan M. Yang X. Gu H. Li Z.. (2020). Incidence of multiple sclerosis in China: a nationwide hospital-based study. Lancet Reg Health West Pac 1:100010. doi: 10.1016/j.lanwpc.2020.100010, PMID: 34327341 PMC8315658

[ref19] Walton C. King R. Rechtman L. Kaye W. Leray E. Marrie R. A.. (2020). Rising prevalence of multiple sclerosis worldwide: insights from the atlas of Ms, third edition. Mult. Scler. J. 26, 1816–1821. doi: 10.1177/1352458520970841, PMID: 33174475 PMC7720355

[ref20] Wang S. Le T. Q. Kurihara N. Chida J. Cisse Y. Yano M.. (2010). Influenza virus-cytokine-protease cycle in the pathogenesis of vascular hyperpermeability in severe influenza. J. Infect. Dis. 202, 991–1001. doi: 10.1086/656044, PMID: 20731583 PMC7537608

[ref9002] Yednock T. A. Cannon C. Fritz L. C. Sanchez-Madrid F. Steinman L. Karin N. (1992). Prevention of experimental autoimmune encephalomyelitis by antibodies against alpha 4 beta 1 integrin. Nature 356, 63–66. doi: 10.1038/356063a0 1538783

[ref21] Zheng J. Wong L. R. Li K. Verma A. K. Ortiz M. E. Wohlford-Lenane C.. (2021). Covid-19 treatments and pathogenesis including anosmia in K18-hace2 mice. Nature 589, 603–607. doi: 10.1038/s41586-020-2943-z, PMID: 33166988 PMC7855185

